# Diagnosis and treatment of hyperinsulinaemic hypoglycaemia and its implications for paediatric endocrinology

**DOI:** 10.1186/s13633-017-0048-8

**Published:** 2017-08-29

**Authors:** Huseyin Demirbilek, Sofia A. Rahman, Gonul Gulal Buyukyilmaz, Khalid Hussain

**Affiliations:** 1 Department of Paediatric Endocrinology, Hacettepe University, Faculty of Medicine, Ankara, Turkey; 2 Great Ormond Street Institute of Child Health, Genetics and Genomic Medicine, University College London, 30 Guilford Street, London, WC1N 1EH UK; 3 Department of Paediatric Medicine Sidra Medical & Research Center, OPC, C6-337, PO Box 26999, Doha, Qatar

**Keywords:** Hyperinsulinaemic hypoglycaemia, Congenital hyperinsulinaemia, Children, Diffuse, Focal, Sirolimus

## Abstract

Glucose homeostasis requires appropriate and synchronous coordination of metabolic events and hormonal activities to keep plasma glucose concentrations in a narrow range of 3.5–5.5 mmol/L. Insulin, the only glucose lowering hormone secreted from pancreatic β-cells, plays the key role in glucose homeostasis. Insulin release from pancreatic β-cells is mainly regulated by intracellular ATP-generating metabolic pathways. Hyperinsulinaemic hypoglycaemia (HH), the most common cause of severe and persistent hypoglycaemia in neonates and children, is the inappropriate secretion of insulin which occurs despite low plasma glucose levels leading to severe and persistent hypoketotic hypoglycaemia. Mutations in 12 different key genes (*ABCC8, KCNJ11, GLUD1, GCK, HADH, SLC16A1, UCP2, HNF4A, HNF1A, HK1, PGM1 and PMM2*) constitute the underlying molecular mechanisms of congenital HH. Since insulin supressess ketogenesis, the alternative energy source to the brain, a prompt diagnosis and immediate management of HH is essential to avoid irreversible hypoglycaemic brain damage in children. Advances in molecular genetics, imaging methods (^18^F–DOPA PET-CT), medical therapy and surgical approach (laparoscopic and open pancreatectomy) have changed the management and improved the outcome of patients with HH. This up to date review article provides a background to the diagnosis, molecular genetics, recent advances and therapeutic options in the field of HH in children.

## Background

Hyperinsulinaemic hypoglycaemia (HH), represents a group of clinically, genetically, and morphologically heterogeneous disorders characterized by inappropriate insulin secretion from pancreatic β-cells [1]. It is the most frequent cause of persistent hypoglycaemia in neonates and infants. Patients with HH have increased risk of permanent brain injury secondary to the metabolic actions of insulin. Insulin drives glucose into insulin sensitive tissues and inhibits endogenous glucose production through the inhibition of glycolysis and gluconeogenesis. The net effect is a decrease in the plasma glucose levels. Insulin also inhibits fatty acid release and ketone body synthesis which are the main alternative fuels of energy for brain neurons during states of low blood glucose [2]. Therefore, it is essential to make a rapid diagnosis of HH and immediately provide appropriate treatement of these patients to prevent the potentially associated neurological complications [3].

The clinical presentation of HH can vary and patients can present either with mild non-specific symptoms of hypoglycaemia (such as poor feeding, lethargy and irritability) or more severe symptoms (such as apnoea, seizures or even coma). HH can be congenital (known as congenital hyperinsulinaemic hypoglycaemia) or secondary to certain risk factors like birth asphyxia, intra-uterine growth retardation [4], maternal diabetes mellitus or associated with various developmental syndromes such as Beckwith-Wiedemann syndrome or metabolic conditions like congenital disorders of glycosylation (CDG) syndromes [5]. Most forms of HH present with fasting hypoglycaemia but in some cases the hypoglycaemia is provoked by protein/leucine loading or even straneous exercise [6–8]. Patients with HH may have an extremely variable clinical phenotype, from completely asymptomatic hypoglycaemia to pharmacologically responsive mild disease whilst in the severe cases, the disease is medically unresponsive and thus the patient requires surgical intervention [1, 9].

At the histological level, congenital forms of HH are classified into three subgroups: diffuse, focal, and atypical forms [1, 10, 11]. Differentiation of histological subtype is closely related to the management and outcome. Nevertheless, the clinical and biochemical fetaures do not differ and require further diagnostic tools. Recent advances in molecular genetics analysis and ^18^F–fluoro-L-dihydroxyphenylalanine (18F–DOPA) PET/CT imaging have fundamentally changed the clinical approach to patients with HH [12, 13].

Mutations in key genes which play a role in the regulation of insulin secretion, constitute the uderlying molecular genetics of congenital HH. Until recently mutations in 9 different genes *(ABCC8, KCNJ11, GLUD1, GCK, HADH*, *SLC16A1, UCP2, HNF4A*and *HNF1A*) that lead to dysregulated secretion of insulin had been described [5, 14, 15]. More recently, abnormalities in three new genes have been linked to HH; *hexokinase 1(HK1), phosphoglucomutase 1 (PGM1)* and *phosphomannosmutase 2 (PMM2)* genes [16–19].

## Regulation of glucose homeostasis

A balance between glucose production (exogenous and/or endogenous) and its utilization is the key to maintain a normal plasma glucose level. In healthy individuals, maintenance of a normal plasma glucose concentration requires a properly functioning hormonal system for integrating and modulating substrate mobilization, interconversion, and utilization. The main regulatory mechanisms are those involved in glucose mediated insulin secretion, glucose generation and utilization. Fatty acid mobilization and metabolism also play a crucial role in the maintenance of glucose homeostasis in infants and children. Beta oxidation of free fatty acids (FFAs) produces ketone bodies which can across the blood-brain barrier and provide the main alternative energy fuel for brain neurons. This would eventually reduce the requirement of glucose, particularly in the fasting state [20]. During fasting, children have a more rapid decline in plasma glucose concentration due to relatively less glycogen stores and a more rapid increase in the plasma concentration of ketone bodies than adults do [21]. In addition, infants are more capable of utilizing the ketones. Therefore children with HH are at a higher risk of brain damage compared to the adults [22, 23].

## Physiological mechanisms regulating insulin secretion from the pancreatic β-cells

**Fig. 1:**
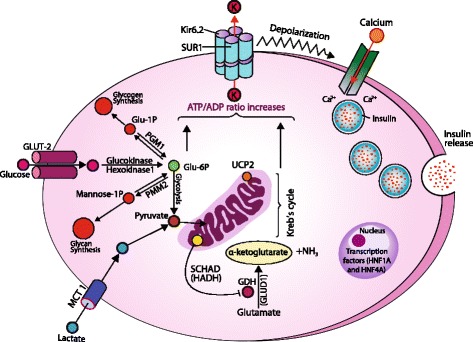
Regulation of insulin release from pancreas β-cell and site of gene mutations involve in the genetics etiology of HH (SUR1: Sulphonyurea receptor 1; Kir 6.2: Inward rectifier potassium channel 6.2; K: Potassium; MCT1: Monocarboxlase transferase-1; Glu: glucose; P: Phosphorus; PGM1: Phosphoglucomutase 1; PMM2: Phosphomannose-mutase 2; UCP2: Mitochondrial uncoupling protein 2; NH_3_: Ammonia; GDH: Glutamate dehydrogenase; GLUD1: Glutamate dehydrogenase 1 gene; HADH: Hydroxy-acyl-CoA dehydrogenase; HNF1A and 4A: Hepatocyte nuclear factor 1 and 4; Ca^+2^: Calcium

The pancreatic β-cell possesses a unique signal transduction system, which links the metabolism of the fuel stimulus to initiate insulin secretion, the so called “stimulus-response coupling” [24]. Glucose is the most important fuel involved in the stimulus-response coupling mechanism. This stimulus response-coupling event is controlled by ATP-sensitive potassium channels (K_ATP_) located in the pancreatic β-cell membrane. Under normal physiological conditions, the metabolism of glucose is intricately linked to insulin secretion from pancreatic β-cells [25]. Glucose enters the β-cell through facilitative glucose transporters, particularly glucose transporter 2 (*GLUT 2*) and is converted to glucose-6-phosphate (G6P) by the enzyme glucokinase [26]. *GLUT2* has high affinity for glucose which allows glucose transport in proportion to the plasma glucose concentrations [27]. Utilization of G6P through glycolysis generates high energy molecules; adenosine triphosphate (ATP) and increases the ratio of ATP/ADP (adenosine diphosphate) which closes the ATP-sensitive potassium channels (K_ATP_). K_ATP_ channels are responsible for the regulation of intracellular and extracellular ion exchange and maintaining a steady state membrane potential. The closure of the K_ATP_ channels results in depolarization of pancreatic β-cell membrane and activation of voltage-gated calcium channels located in the β-cell membrane. Calcium enters into β-cell through these voltage-gated calcium channels and the increase in intracellular calcium triggers insulin secretory granule exocytosis (Fig. 1).

Glucokinase also plays a critical role in acting as a gluco-sensor, providing a link between the extracellular plasma glucose concentration and the metabolism of glucose in the β-cell [28]. When the plasma glucose concentration is increased, the activity of glucokinase is also increased, hence increasing insulin production and secretion from the β-cell (Fig. 1). Similarly, as the plasma glucose concentration decreases, serum insulin becomes undetectable when plasma glucose concentrations fall below 3 mmol/l [29, 30].

## Transient (causes of) hyperinsulinaemic hypoglycaemia

In babies with HH that resolves spontaneously either in a few days or weeks postpartum, transient HH is diagnosed. Transient HH can develop secondary to maternal diabetes mellitus, the use of intravenous dextrose given during labour, as well as intrauterine growth restriction (IUGR), small baby for gestational age (SGA) and perinatal asphyxia. Prolonged transient HH in IUGR and asphyxia babies have been reported to require treatment with diazoxide [31]. However, patients have also been noted to present with transient HH in the absence of the aforementioned risk factors [32]. The mechanism(s) behind this still remains unknown.

## Congenital hyperinsulinaemic hypoglycaemia

The genetic basis of congenital HH involve defects in the pathways that regulate insulin release. These defects cause disturbances in the glucose-mediated insulin secretion processes and inappropriate release of insulin from the pancreatic β-cells. This therefore leads to severe hypoglycaemia in children. Currently, mutations in 12 genes have been reported to cause congenital HH. However, the underlying molecular genetics etiology that causes congenital HH still remains unknown in about 50% of patients. Table 1 outlines the transient and permanent causes of HH.

**Table 1:** Transient and permanent causes of hyperinsulinaemic hypoglycaemia

1. Transient causes of HH • Maternal diabetes mellitus (gestational and insulin-dependent) • Use of maternal intravenous dextrose during labour • Intrauterine growth restriction • Perinatal asphyxia • Rhesus isoimmunisation	
2. Genetic causes of HH • Channelopathies ○ ABCC8 ○ KCNJ11 • Metabolopathies ○ GLUD1 ○ HADH ○ GCK ○ SLC16A1 ○ HNF1A ○ HNF4A ○ UCP2 ○ HK1 ○ PGM1 ○ PMM2	
3. Metabolic causes of HH • Congenital disorders of glycosylation • Tyrosinaemia type 1	
4. Syndromic causes of HH • Beckwith-Wiedemann • Kabuki • Trisomy 13 • Central hypoventilation syndrome • Leprechaunism (insulin resistance syndrome) • Mosaic Turner • Sotos • Usher • Timothy • Costello	
5. Miscellaneous causes of HH • Postprandial HH ▪ Insulin gene receptor mutation ▪ Dumping syndrome ▪ Noninsulinoma pancreatogenous hypoglycaemia syndrome (adults) ▪ Insulin autoimmune syndrome (mostly adults) ▪ Bariatric surgery (adults) ▪ Insulinoma • Non-islet cell tumour hypoglycaemia (adults) • Factitious hypoglycaemia • Drug-induced	

## Genetics of Hyperinsulinaemic Hypoglycaemia

### Pancreatic β-cell K_ATP_ channel defects

The most prevalent and serious genetic cause of congenital HH is due to defects in the genes encoding the K_ATP_ channel (also known as channelopathies) [33, 34]. The K_ATP_ channels are made up of two protein subunits; the sulphonlyurea receptor 1 (SUR1), which is encoded by the *ATP Binding Cassette Subfamily C Member 8* (*ABCC8)* gene, and the inwardly rectifying potassium (Kir6.2), encoded by the *Potassium Voltage-Gated Channel Subfamily J Member 11 (KCNJ11)* gene. Both these genes are located on chrosome11p15.1. *ABCC8* is 39 exons in length, whilst the *KCNJ11* gene is only made up of 1 exon [35]. The K_ATP_ channel complex is composed of four outer SUR1 subunits and four inner (pore-making) Kir6.2 proteins. The SUR1 component regulates the activity of the Kir6.2 proteins, in addition to a role in initiating a response and sensitivity to sulphonylurea drugs and K_ATP_ channel opening drugs such as diazoxide [36, 37]. The inner Kir6.2 proteins allow influx of potassium ion concentrations across the membrane. Intracellular nucleotides such as ADP and ATP mediate the action of the K_ATP_ channel. A change in the ratio of ATP to ADP in the β-cell causes closure of the K_ATP_ channel and triggers depolarisation of the cell membrane and activates the voltage-gated calcium channels [38]. This in turn causes insulin release through exocytosis and thus promotes lowering of plasma glucose [39].

Recessive inactivating (loss-of-function) K_ATP_ channel gene mutations predominantly cause medically unresponsive diffuse congenital HH, while, mild -even adult-onset- HH have also been reported [5, 40–42]. Autosomal dominant inherited mutations usually cause milder forms of congenital HH, whilst, medically unressponsive forms have also been reported [43, 44].

Another ten gene defects; *GLUD1, HADH, GCK, SLC16A1, HNF1A, HNF4A*, *UCP2, HK1, PGM1*and *PMM2* have been proposed to cause congenital HH [19, 45]. Known as the metabolopathies due to mutations resulting in defects in the metabolic pathways that cause unregulated insulin release and associated hypoglycaemia.

### Glutamate dehydrogenase (GLUD1) mutations and Hyperinsulinism-hyperammonaemia syndrome (HI/HA)

During states of hypoglycaemia, alternative fuel sources are required. The *Glutamate Dehydrogenase 1* (*GLUD1)* gene encodes for the mitochondrial enzyme; glutamate dehydrogenase (GDH) which is essential in glutamate metabolism and has an important role in generating amino acids as fuel substitutes in the absence of glucose [6]. GDH appears to be sensitive to the amino acid leucine and nucleotide phosphate potential [7]. Both of these regulate GDH activity and thus stimulate amino acid-mediated insulin release. The *GLUD1* mutations are associated with recurrent fasting and postprandial HH and is also known as the hyperinsulinism/hyperammonaemia (HI/HA) syndrome because of features that include high serum ammonia (usually two-to-three-times the upper normal range) after consumption of a high protein meal. These infants usually are not lethargic/comatose as occurs in inborn errors of metabolism with hyperammonaemia, therefore, unless the serum ammonia level, the diagnosis of HI/HA syndrome may be missed. Urinary α-ketoglutarate is elevated in patients with HI/HA syndrome [46].

*GLUD1* mutations account for the most prevalent metabolopathic cause of congenital HH. Studies to date have identified mutations in exons 6, 7, 11 and 12 in the 13 exon gene to cause congenital HH [47, 48]. Although *GLUD1* activating mutations occur mainly as de novo, some have reported patients presenting with autosomal dominant forms also [48, 49]. Since K_ATP_ channels are unaffected by the mutations, patients are medically controlled by the channel activator; diazoxide and are maintained on a low protein diet. Patients carrying *GLUD1* mutations have been reported to be more sensitive in developing epileptic seizures irrespective of the severity and frequency of hypoglycaemia episodes. This has been attributed to the shift in the glutamate metabolism leading to a decline in the inhibitory neurotransmitter γ-aminobutyric acid (GABA) [50].

### Hydroxyacyl-CoA dehydrogenase (HADH) mutations and congenital HH

Hydroxyacyl-CoA dehydrogenase (HADH) or short chain L-3-hydroxyacyl-CoA dehydrogenase (SCHAD) is another mitochondrial enzyme that oxidises fatty acids to produce ATP. Although this gene is expressed in the liver, kidneys, muscle and heart, it is most abundant in the pancreatic islets [51]. The *HADH* gene has 8 exonic regions and mutations inherited in an autosomal recessive manner appears to reduce the enzymes inhibitory action on GDH [52–54]. Therefore, *GLUD1* and *HADH* patients present similarly with protein sensitivity, although *GLUD1* mutations also cause hyperammonemia. *HADH* gene mutations can lead either to severe neonatal congenital HH or to mild late-even adult-onset, protein–induced HH which is usually diazoxide responsive [55, 56]. Although the clinical and etiological relevance are not clear, patient with *HADH* mutations may have elevated plasma concentrations of 3-hydroxy-butyryl-carnitine and urinary 3-hydroxy-glutarate [54, 57].

### Glucokinase (GCK) mutations and congenital HH

The *GCK* gene is 12 exons long and encodes the enzyme; glucokinase (GCK). *GCK* can be found in the pancreatic β-cells, liver and brain [58]. The GCK protein, catalyses glucose to G6P as substrate for glycolytic pathway which generates ATP, therefore, is essential in the glucose-dependent insulin release. The GCK enzyme has high affinity to glucose, serving as a glucose-sensor in pancreatic β-cells. Dominant activating mutations in *GCK* cause alteration in the protein structure and functions from having a low affinity for glucose to having a high affinity and reduces the threshold for glucose-stimulated insulin release [59, 60]. Variable clinical presentations occur ranging from neonatal severe HH to mild hypoglycaemia or even adult-onset asymptomatic or severe congenital HH, majority of which respond to diazoxide whilst some require surgery [61–65].

### Exercise-induced Hyperinsulinaemic Hypoglycaemia

The *Solute Carrier Family 16 Member 1 (SLC16A1)* gene encodes for the monocarboxylate transporter (MCT1). This protein has a role in transporting monocarboxylates such as pyruvate or lactate into the Krebs cycle to produce ATP and promote insulin release independently of glucose [66]. However, the low expression of this gene in the normal pancreatic β-cell suggests a physiological response to ensure against HH. Autosomal dominant gain-of-function mutations in *SLC16A1* cause the expression of MCT1 in β-cells. This in turn leads to glycolysis-generated pyruvate to continually enter the Krebs cycle and induces inappropriate insulin secretion in states of low plasma glucose during anaerobic exercise [67]. Congenital HH due to these mutations is also known as exercise-induced HH, since vigorous exercise stimulates hypoglycaemia. Diagnostically, *SLC16A1* patients present with HH after a pyruvate load test [68]. Typically patients with exercise-induced HH develop hypoglycaemia following a bout of strenous exercise. Some patients may require therapy with diazoxide while others can be mananged with diet [8].

### Hepatocyte nuclear factor 1A&4A (HNF1A&4A) and congenital HH

The hepatocyte nuclear factors 1A and 4A genes (*HNF1A/HNF4A*) encode for the HNF1*-a* and HNF4-*a* proteins, respectively. These are transcription factors for nuclear hormone receptors expressed in pancreatic β-cells and regulates glucose-dependent insulin secretion [69, 70]. Inactivating mutations in *HNF1A* and *HNF4A* causes congenital HH in children and then maturity-onset diabetes of youth (MODY type 3 in *HNF1A* and type 1 in *HNF4A*) [71–73].

*Hnf1a* null mice have unregulated insulin release, but the mechanism for this remains yet to be determined [74]. The exact role of HNF4α in insulin regulation also is unknown since studies have found conflicting results using transgenic *Hnf4a* mouse models [69, 75]. Another potential explanation for this disease-causing mutation is the loss of peroxisome proliferators-activated receptor alpha (PPAR alpha; another nuclear factor). PPARα’s role appears to direct fatty acids to the β-oxidation pathway and thus lead to hyperinsulinism during glucose depletion [76]. Moreover, in support of this, the *Hnf4* alpha null mice have a decrease in PPARα expression, whilst the *Ppar*α knockout mice have been shown to develop fasting hypoglycaemia [75, 77].

Mutations in both *HNF1A* and *HNF4A* have been identified in macrosomic births and can range from mild transient to severe and permanent HH [5, 42, 71, 78]. Patients generally respond to diazoxide and HH resolves with age [5, 42, 79, 80]. Mutation in the *HNF4A* gene have been reported in cases with HH, increased level of glycogen in erythrocytes, elevated liver transaminases, and increased echogenicity on liver ultrasonography suggesting glycogenosis-like phenotype [81, 82]. Although, thought to be rare causes of congenital HH, mutations in *HNF1A* and *HNF4A* have been reported at higher incidences in some series, particularly in those have diazoxide-responsive HH [79, 83].

### Mutations in the uncoupling protein 2(UCP2) gene and congenital HH

Uncoupling protein 2 (UCP2) is mitochondrial carrier protein and encodes for the *UCP2* gene. Expressed in the pancreatic α- and β-cells, UCP2 inhibits glucose oxidation and activates glutamine oxidation by carrying four-carbon metabolites out of the mitochondria [84]. Inactivation of the *UCP2* gene would therefore, promote glucose oxidation and thus cause HH. Initially, it appeared that *UCP2* causing HH would resolve with age suggestive of a transient nature since the original cases were reported in patients of up to 6 years, however, new cases have emerged where HH continued beyond 10 years of age [85–87]. In a recent study, *UCP2* mutations were reported for the underlying molecular genetics of 2.4% in a cohort of 211 patients with diazoxide-responsive congenital HH [87]. These patients developed hypoketotic-hypoglycaemia after a fairly good fasting tolerance, while some developed symptomatic hypoglycaemia following an oral glucose load. Rapid decline in plasma glucose and inappropriate insulin secretion following glucose load suggested that mutations in *UCP2* cause glucose-induced HH rather than fasting hypoglycaemia [87].

### Hexokinase 1 (HK1) gene mutations and congenital HH

*HK1* is located on chromosome 10 and encodes the enzyme; hexokinase 1 (HK1). HK1 is one of the key enzymes involved in regulating glucose homeostasis. Normally, *HK1* expression is silenced in the pancreatic β-cells. However recently, a report identified a dominant gain-of-function mutation in the *HK1* gene to cause HH in a family with “idiopathic hypoglycaemia of infancy” [16, 86].

### Phosphoglucomutase 1 (PGM1) gene mutations and congenital HH

Phosphoglucomutase 1 (PGM1) is involved in glycogen metabolism and a loss-of-function mutation in the *PGM1* gene has been shown to be associated with hypoglycaemia [17]. Patients with these inactivating mutations have presented with fasting hyperketotic hypoglycaemia, as well as postprandial HH [18].

### Phosphomannomutase 2(PMM2) gene mutations and congenital HH

The *phosphomannomutase 2 gene* (*PMM2*) has recently been reported to cause HH as well as congenital polycystic kidney disease in 17 children from 11 unrelated families [19]. The group reported a promoter mutation (c.-167G > T) in the *PMM2* gene in all affected patients. *PMM2* encodes for an enzyme in glycosylation, deglycosylation in pancreatic β-cells has been shown altered insulin secretion.

## Clinical presentation of hyperinsulinaemic hypoglycaemia

HH can produce a variety of symptoms and effects, ranging from non-specific adrenergic symptoms (poor feeding, hunger, palpitation, sweating) to life-threatening neuroglycopenic symptoms arising from an inadequate supply of glucose to the brain neurons resulting in impairment of brain functions (seizures, unconsciousness, lethargy, coma, and even death). Delay in the diagnosis and inappropriate management from prolonged and recurring hypoglycaemia my result in permanent neurological sequelae. The neurological outcome is closely related to the severity, duration as well as the underlying mechanism for hypoglycaemia. In particular, disorders affecting both ketogenesis and hypoglycaemia have higher risk of permanent brain damage [88–90]. Newborns with congenital HH may be macrosomic due to intrauterine hyperinsulinaemia, however; the absence of macrosomia does not exclude HH.

HH most commonly presents in the newborn, but it can also present during infancy, childhood and even adulthood [40, 87]. The clinical presentation of hypoglycaemia is most severe in the newborn and may be quite subtle in the infancy and childhood periods [91]. However, some are not diagnosed until the first year or during childhood [91–93]. Even within the same family, the severity of disease can vary substantially. In a review of 114 cases, 65% presented as neonates, 28% as infants, and 7% during childhood [91]. Congenital HH caused by some genetic defects may be mild and in some cases resolves spontaneously. This was seen in a case series of infants with congenital HH but with normal K_ATP_ channel and glutamate dehydrogenase function [94].

The HH due to recessive mutations in *ABCC8/KCNJ11* genes is usually refractory to oral feeds and requires high concentrations of intravenous glucose to maintain normoglycaemia [3]. However, in the milder forms patients may be able to maintain normoglycaemia on oral feeds.

Hypertrophic cardiomyopathy and hepatomegaly (increased storage of glucose as glycogen) are observed in some patients with HH. The mechanism of cardiomyopathy and hepatomegaly in these patients is unclear but might be related to the effect of foetal hyperinsulinaemia [3].

## Diagnosis and investigations of HH

The early diagnosis of HH is fundamentally important for preventing hypoglycaemic brain injury, hence, clinicians should always be aware of recognising and managing these patients. Any patient with recurrent or persistent hypoglycaemia can potentially have HH, and this is the only cause of hypoglycaemia which persists despite continuous administration of glucose. A powerful clue to the dysregulated insulin secretion is the high intravenous glucose infusion rate required to maintain normoglycaemia. An intravenous glucose infusion rate of >8 mg/kg/min (normally is 4–6 mg/kg/min) is virtually diagnostic of HH [3].

In milder forms of HH, it will be important to establish the duration of fasting and whether the hypoglycaemia is precipitated by meals (protein sensitivity) or by exercise.

In HH, there is an inappropriate concentration of serum insulin/c-peptide for the low level of plasma glucose (spontaneous or provoked). The metabolic effect of inappropriate insulin secretion is reflected by the inappropriately low levels of serum ketone bodies and fatty acids during the hypoglycaemic episode. There is no correlation between the serum insulin concentration and the severity of the hypoglycaemia [95]. In some difficult cases, the diagnosis of HH should not be based on an isolated serum insulin/c-peptide concentration but on the clinical presentation and the biochemical profile of insulin action (low β-hydroxybutyrate and fatty acid concentrations). The diagnostic criteria for HH are summarised in Table 2.

**Table 2:** Diagnostic criteria for patients with HH [1, 101]

Diagnostic criteria	• Plasma glucose <3 mmol/l with: • Detectable serum insulin • Detectable C-peptide (In endogenous HH) • Suppressed/low serum ketone bodies • Suppressed/low serum fatty acids
Supportive evidences (when diagnosis is in doubt or difficult):	• Glucose infusion rate > 8 mg/kg/min • Positive glycaemic (>1.5 mmol/L) response to intramuscular/ intravenous glucagon • Positive glycaemic response to a subcutaneous/intravenous dose of octreotide • Low serum levels of IGFBP1 [insulin negatively regulates the expression of IGFBP1] • Suppressed branch chain (leucine, isoleucine and valine) amino acids • Provocation tests (leucine loading or exercise testing) may be needed in some patients • Supressed/undetectable urine ketone • Normal lactic acid • Normal plasma hydroxybutyrylcarnitine^a^ • Normal ammonia^b^ • Appropriate counterregulatory hormone response^c^ -Cortisol > 20 mcg/dl (500 nmol/l) -Growth hormone > 7 ng/ml In the neonatal period if the hypoglycaemia persists for >48 h this will require investigation

^a^Elevated in HH due to HADH gene mutation, ^b^Elevated in hyperinsulinism-hyperammonemia syndrome (HI-HA) due to *GLUD1* gene mutation, ^c^Counterregulatory hormone response may be blunted in spontaneous, particularly recurring hypoglycaemia

An elevated serum ammonia concentration (appropriately collected and analysed) in a patient with HH is suggestive of the hyperinsulinism and hyperammonaemia (HI/HA) syndrome [47]. Raised plasma hydroxybutyrylcarnitine and urinary 3-hydroxyglutarate are diagnostic of a rare type of congenital HH (hydroxyacyl-Coenzyme A dehydrogenase (*HADH*) deficiency) [57].

Some types of HH are elicited only after provocation testing. For example in patients who have the HI/HA syndrome (who have fasting as well as protein induced hypoglycaemia) protein/leucine loading precipitates hypoglycaemia [96]. The patients with exercise-induced HH will require a formal exercise test and or a pyruvate load to demonstrate post exercise induced HH [8, 68]. In some patients a positive glycaemic response (rise in the plasma glucose concentration of >1.5 mmol/L) following an intramuscular/intravenous injection of glucagon at the time of hypoglycaemia provides supportive evidence [97]. A glycaemic response to a subcutaneous dose of octreotide may also aid diagnosis. On the other hand as insulin suppresses the transcription of the *insulin-like growth factor binding protein 1 (IGFBP-1)* gene thereby IGFBP-1 synthesis serum levels of (IGFBP-1) is decreased in HH [98].

## Hyperinsulinaemic hypoglycaemia management

Patients with HH are at risk of severe brain injury, leading to short-term acute neurological symptoms (eg, seizure, lethargy, coma) and long-term neurological sequelae (eg, epilepsy, cognitive deficits, microcephaly) [99]. Hence, prompt diagnosis and immediate treatment is essential to avoid such complications.

Treatment of HH includes medical, surgical or sometimes combination therapies. Increasing and maintaining the plasma glucose concentration at a safe range (above 3.5 mmol/L) is the goal of emergency therapy. The principal of long-term therapy is setting an equilibrium by inhibiting inappropriate insulin secretion and/or providing adequate glucose supply [100].

## Emergency management

The goal of emergency treatment is to achieve normoglycemia immediately, keep plasma glucose levels at a safe range (>3.5 mmol/L) while the etiological investigations for differential diagnosis and long-term treatment planing are in progress.

### Parenteral glucose infusion

At the time of hypoglycemia, once the critical sample has been obtained, if hypoglycemia is unresponsive to oral feeds, or the patient is unable to take an oral feed, a mini bolus of 2mls/kg 10% glucose should be administered intravenously over 1 min. To achieve normoglycemia and keep plasma glucose within the safe range, a continuous intravenous glucose infusion of 6–8 mg/kg/min should be commenced immediately. Patients with HH usually require a very high glucose infusion rate (GIR) to achieve and maintain normoglycaemia. Therefore, GIR might be adjusted according to consecutive plasma glucose measurements.

### Glucagon administration

Glucagon induces glycogenolysis, gluconeogenesis, ketogenesis and lipolysis, and may be used in emergency situations where patients are unable to take oral feed and/or venous access is difficult to obtain [101, 102]. The recommended single dose is between 0.5–1 mg [103]. Glucagon, in high doses, may cause rebound hypoglycemia due to a paradoxical increase in insulin secretion [104]. Glucagon can also be administered as continuous intravenous/subcutaneous infusion at a rate of 5-10mcg/kg/h for short-term stabilization of plasma glucose as well as long-term non-surgical management of congenital HH, in combination with octreotide [105, 106].

### Frequent feeding

In infants, frequent high calorie carbohydrate feeds may reduce the frequency and severity of hypoglycaemic episodes. As these patients usually have food aversion, a percutaneous gastrostomy (PEG) may help to feed frequently and provide an opportunity to administer enteral boluses of high calorie-carbohydrate solutions [107, 108]. Uncooked cornstarch may help to decrease the hypoglycaemic episodes, particularly to improve fasting tolerance during a prolonged overnight fast in children over the age of one year.

## Long-term management

The principles of long term management are inhibiting insulin secretion, thereby prevent recurrent hypoglycaemia episodes, provide an age appropriate fasting tolerance and avoid neurological symptoms associated to hypoglycemia.

### Diazoxide

Diazoxide, the first-line drug for HH, binds to and opens the SUR1 subunit of the K_ATP_ channel [101, 102, 109, 110]. Diazoxide is usually effective in all forms of congenital HH, except severe, neonatal-onset, recessive, and focal forms caused by mutations in the *ABCC8* and *KCNJ11* [109]. Diazoxide needs an intact K_ATP_ channel activity to work properly. Therefore, diazoxide responsiveness has been the key point for the molecular genetics analysis, differential diagnosis and management strategies of HH. If a patient is unresponsive to the diazoxide, further genetic analysis for *ABCC8/KCNJ11* and ^18^F–DOPA-PET/CT scan must be performed to differentiate histological subtypes, focal or diffuse disease. The initial dose for diazoxide is usually 5 mg/kg/day, in 3 divided doses and then gradually can be increased up to maximum dose of 15–20 mg/kg/day [111]. The dose should be adjusted to achieve appropriate fasting tolerance for age and normoglycemia under a normal feeding plan. Fluid retention, hypertrichosis and feeding problems are the most common side effects of diazoxide. Therefore, especially in the newborns, when trying to reduce the risk of fluid retention, a thiazide diuretic, chlorothiazide (7–10 mg/kg/day in 2 divided doses), should be administered with diazoxide. Hyperuricaemia, tachycardia and leukopenia, are other rare side effects [112] (Table 3). During follow up, if the diazoxide dose required to maintain normoglycaemia is lower than 5 mg/kg/day, consideration should be given to possibly withdrawing diazoxide in the hospital setting as some types of HH gradually resolve over time [113].

**Table 3:** Drugs for medical therapy of hyperinsulinaemic hypoglycaemia [1, 110, 113]

	Route	Dose	Mode of action	Side effects
Conventional medicines				
Diazoxide	*Oral*	5–20 mg/kg/day, in 3 divided doses	Bind to SUR1 subunit of K_ATP_channels, opens the channels and inhibits insulin secretion Requires an intact K_ATP_ channel activity to work properly	Common: Water and salt retention, hypertrichosis, loss of appetite Rare: Cardiac failure, hyperuricaemia, blood dyscrasias (bone marrow suppression, anaemia, eosinophilia etc.), paradoxical hypoglycaemia
Chlorothiazide	Oral	7–10 mg/kg/day, in 2 divided doses	Prevents fluid retention, synergistic effects with diazoxide on K_ATP_ channels to inhibit insulin secretion	Hyponatraemia, hypokalaemia
Nifedipine	Oral	0.25–2.5 mg/kg/day, in 2–3 divided doses	Inhibits Ca-channels of the β-cell membrane	Hypotension
Octreotide	s.c	5–35 μg/kg/day, divided to 3–4 doses or continuous subcutaneous infusion	Activation of SSTR-2 and SSTR-5 inhibits calcium mobilization and acetylcholine activity, and decreases insulin gene promoter activity, reduces insulin biosynthesis and insulin secretion.	Acute: Anorexia, nausea, abdominal discomfort, diarrhoea, drug induced hepatitis, elevated liver enzyme, long QT syndrome, tachyphylaxis, necrotizing enterocolitis Long-term: Decreases intestinal motility, bile sludge and gallstone, suppression of pituitary hormones (Growth hormone, TSH)
Glucagon	s.c/i.m bolus or s.c/i.v infusion	0.02 mg/kg/dose or 5–10 μg/kg/h infusion	G-protein coupled activation of adenylate cyclase, increases cAMP, Induces glycogenolysis and gluconeogenesis	Nausea, vomiting, skin rash and rebound hypoglycaemia in high doses (>20 μg/kg/h) due to paradoxical activation of insulin secretion
New medicines				
Rapamycin (sirolimus, everolimus)	Oral	An initial dose of 1 mg/m2 per day may require dose adjustment according to blood sirolimus concentration usually to keep between 5 and 15 ng/ml	mTOR inhibitor. Inhibits insulin release and β-cell proliferation through different mechanism which have not been clarified yet	Immune suppression, mucositis, hyperlipidemia, elevation of liver enzyme, thrombocytosis, impaired immune response to BCG vaccine
Octreotide LAR/ Lanreotide	Deep s.c	Total 4 weekly dose of octreotide given every 4 weekly or 15–60 mg/every 4 weekly	These long acting somatostatin analogues have similar effects as daily multidose octreotide.	Similar to daily multiple injection octreotide. However, long-term follow up is not known yet

### Octreotide

Octreotide, the second line drug of HH, is an 8 amino acid synthetic somatostatin analogue that inhibits insulin secretion by binding to somatostatin receptor 2 and 5 (SSTR2 and SSTR5) [114]. Activation of SSTR5 decreases the insulin gene promoter activity and inhibits calcium mobilization and acetylcholine activity [115]. Somatostatin also inhibits the K_ATP_ channel which results in reduced insulin secretion [102]. The initial dose of octreotide is 5 μg/kg/day given by subcutaneous injections at 6–8 h intervals. The recommended maximum dose is 30–35 μg/kg/ day. Long-term continuous, subcutaneous octreotide infusion with an insulin pump has also been reported as a feasible alternative to surgery for patients with monoallelic K_ATP_-channel mutations [116]. The first response to octreotide administration is usually hyperglycaemia followed by a blunted effect within 24–48 h (tachyphylaxis), thereby, dose adjustment may be required to keep plasma glucose level at normoglycaemic levels [101, 117, 118]. Tachyphylaxis, necrotizing enterocolitis, anorexia, nausea, abdominal pain, diarrhoea, gall bladder pathologies, elevation of liver transaminases, pituitary hormone suppression, long QT syndrome and arrest in linear growth are the reported side effects in patients treated with octreotide [110, 119–126] (Table 3). However, in recent studies evaluating the long-term effects of octreotide therapy in patients with congenital HH the effect of octreotide on linear growth have been found to be of no clinical relevance [110, 121].

### Long-acting somatostatin analogs

Recently long-acting somatostatin analogs have been an effective option in the management of congenital HH. Octreotide long-acting release (LAR) is formulated with biodegradable microspheres [127]. This formulation provides the advantage of administration every 28 days. Lanreotide is also a synthetic octapeptide and it is recommended to inject every 28 days. LAR-octreotide and lanreotide have been successfully used in children with congenital HH [110, 128–132]. The opportunity to administer LAR once in every 4 week not only increases treatment compliance but also increases quality of life (QoL) [128]. Therefore, increasing experiences with LAR may improve the treatment compliance and most favorable long-term outcome of patients with congenital HH.

### Nifedipine

Nifedipine, a calcium channel blocker, inhibits insulin secretion by inactivating the voltage-gated calcium channels [133]. Recommended dose is 0.25–2.5 mg/kg/day divided into 2–3 doses [102]. Hypotension is an uncommon side-effect [102] especially at doses above 0.5 mg/kg/day [134] (Table 3). Although, in many case reports nifedipine treatment has shown to have a favourable effect on HH, in a recent study exclusively investigating its use in HH due to mutations in the *ABCC8* gene, HH has shown not responded nifedipine therapy [135]. It is, therefore, suggested that, mutations in the K_ATP_ channel genes might render the L-type calcium channel ineffective to therapy with nifedipine [135–139].

## New and potential future therapies

A group of patients with diffuse and focal disease are unresponsive to conventional medical therapies, both diazoxide and octreotide. Surgery has been the treatment option for this patients. Nevertheless, after surgery recurrent HH can continue or may be complicated with diabetes mellitus or exocrine pancreas insufficiency. This group of patients are area of interest for clinicians for trial of novel therapeutic options to better improve their short- and long-term outcome.

### Sirolimus

Sirolimus is a mammalian target of rapamycin (mTOR) inhibitor. mTOR is a serine and threonine protein kinase and regulates cellular growth by stimulating protein synthesis by increasing mRNA translation initiation and the capacity of the ribosomal protein machinery [140]. The mechanism of action for mTOR inhibitors in HH has not been fully elucidated. However it is reported that there is constitutive activation and overexpression of p-mTOR on the plasmalemmal aspect of the acinar cells, and activation on the plasmalemmal aspect of the ductal cells in the diffuse variant of congenital HH [141].

A number of autocrine growth factors like IGF-I, vascular endothelial growth factor (VEGF), and epidermal growth factor receptor (ErbB), as well as glucose, fatty acids, and amino acids activate mTOR pathway. Upregulation of mTOR leads increased insulin production in the pancreatic β-cells [142]. Conversely, inhibition of mTOR with rapamycin inhibits insulin secretion as well as β-cell growth [143]. Sirolimus can also induces β-cell apoptosis and promotes insulin resistance. Furthermore, mTORC1 inhibits PPARα-mediated expression of ketogenic genes thereby ketone body synthesis, therefore, down-regulation of the mTOR pathway may restores ketogenesis which inturn reduces the risk of brain injury in patients with HH [21]. Recently, it has been reported that sirolimus is effective and safe for the severe, diazoxide unresponsive diffuse congenital HH with no major side effects [144]. Following the first experience, several number of case reports indicating the successful use of sirolimus have been published [145–149].

Although the optimal therapeutic blood levels for sirolimus in congenital HH patient have not been determined, ranges previously defined for the use of renal transplant patients, 5–15 ng/ml is advised [144]. The most reported adverse effects are stomatitis, increased risk of infection, immunosuppression, renal dysfunction, fatigue, pneumonitis and increased serum aminotransferase or lipid levels [150]. Since, sirolimus have severe side effects, particularly due to risk arising from immunesuppression, patients under sirolimus therapy, should be closely monitored for adverse effects. Despite limited clinical experience in CHI, mTOR inhibitors are thought to be a good option for selected patients who do not respond to diazoxide or octreotide. However, in a recent report evaluating the efficacy of sirolimus in 10 patients with diazoxide unresponsive congenital HH, mTOR inhibition has shown to achieve euglycemia, improve fasting tolerance, and reduced requirement for medical therapy in only three patients (30%) with certain side effects [151]. In addition, pancreatic tissue from two patients who did not respond to sirolimus showed no reduction in cell proliferation, further suggesting that mTOR signaling did not down-regulate proliferation in the pancreas of patients with congenital HH [151]. Nevertheless, as recommended the last option prior to surgery, trying sirolimus before performing surgical therapy may help avoiding from much more severe and lifelong complications of surgery. Even so, this does not eliminate the requirement for further insights into identifying the long-term adverse effects and efficacy of mTOR inhibitors.

#### GLP-1 receptor antagonist: Exendin (9–39)

Glucagon-like peptide-1 (GLP-1), released after a meal, is an incretin hormone produced in enteroendocrine L-cells of the intestine in response to ingested nutrients [152]. GLP-1 stimulates insulin secretion by binding to a guanine nucleotide binding protein-coupled receptor [153], resulting in the activation of adenylate cyclase and generation of cAMP [154]. GLP-1 stimulates insulin secretion by both protein kinase A-dependent and -independent mechanisms [155] and also inhibits glucagon secretion, hepatic glucose production, gastric emptying, and appetite. Exendin-(9–39) is a specific GLP-1 receptor antagonist in mice and humans [156, 157]. A previous study in *Sur-1* knock-out mouse showed that Exendin(9–39) raised fasting plasma glucose levels by decreasing cAMP levels and insulin secretion [158]. Another study demonstrated that exendin-(9–39) prevents hypoglycemia and maintains stability of plasma glucose during a prolonged fast in individuals with K_ATP_-HI [159]. These promising results point to the GLP-1 receptor as a therapeutic target for K_ATP_-HI.

#### Use of chaperones

Mutations in *ABCC8* encoding SUR1 subunit of the K_ATP_ channel inhibit trafficking of channel proteins from the endoplasmic reticulum to the cell surface, thereby, preventing the channel function. Chaperones are small molecules that correct the trafficking of K_ATP_-channels. In previous in-vitro studies, sulfonylureas and carbamazepine, have been shown to correct channel trafficking defects in the transmembrane domains 0 and 1 (TMD0, TMD1) mutations of *ABCC8* [160, 161].

In a recent study, 13 novel *SUR1* mutations that cause channel trafficking defects, were pharmacologically rescued using glibenclamide and carbamazepine [162]. In the cross-linking experiments it was shown that while K_ATP_ channel inhibitors promoted interactions between the N terminus of Kir6.2 and SUR1, channel openers did not. This was suggested that to correct the channel biogenesis and trafficking defects, K_ATP_ channel inhibitors enhance interactions between Kir6.2 and SUR1 subunits [162]. Furthermore, in the functional studies most mutant channels rescued to the cell surface using chaperons exhibited WT-like sensitivity to ATP, MgADP, and diazoxide. After rescuing the trafficking defect diazoxide had effectively recovered the channel function [162]. Although it is still at experimental level and requires further investigations, using chaperones for correction of channel trafficking would perhaps improve diazoxide responsiveness in congenital HH due to *ABCC8* mutations.

#### Ketogenic diet

During the suckling period, ketone bodies constitute a higher proportion of fuel for energy for the immature brain. Upon brain maturation and weaning, blood levels of ketone bodies decrease and glucose becomes the main energy for the mature brain [163]. Increase in ketone bodies concentration promotes the oxidation rate in the brain [164, 165]. For this reason they have been used as therapy against refractory epilepsy and in experimental models of ischemia and excitotoxicity [166]. HH induces severe neuroglycopenia and also inhibits gluconeogenesis, glycogenolysis, lipolysis, and eventually fatty acid oxidation which result in inapproriate ketone synthesis. Therefore, neurological risk of hypoglycaemia further increases because of lack of ketones. In a recent study ketogenic diet was administered in a child with drug-resistant congenital HH due to a spontaneous *GCK* activating mutation for 2 years. After the first six months, the patient had become free of epileptic seizures, with normalization of EEG, and showed a marked recovery in psychological development and quality of life [167]. Although requires further evidence, these findings suggested that ketogenic diet could have a neuroprotective effect despite persistence of neuroglycopenia and it can be used in selected cases of HH.

## Histologic subtypes of congenital HH

**Fig. 2:**
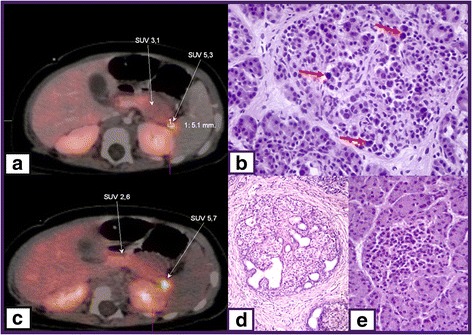
^18^F–DOPA-PET/CT scan images of focal CHI (**a** and **c**), histological figure of diffuse (**b**) and focal (**d**) disease and normal pancreas islet cell (**e**). SUV 5.3 and SUV 5.7 indicate focal uptake of ^18^F–DOPA, red arrows show large nuclei of β-cell in diffuse disease

At the histologic level, there are three forms of congenital HH; focal, diffuse, and atypical disease (Fig. 2). Differentiation of histologic subtypes is essential for the success of surgery.

### Focal form

The abnormal pancreatic β-cells are localised to a specific location in pancreas. Focal pancreatic lesions are generally 2-10 mm in size and appear as small regions of islet adenomatosis (nodular hyperplasia of islet-like cell clusters, including ductuloinsular complexes, Fig. 2d) [1]. Islet cells of particular lesion have large cytoplasm with dispersed abnormal nuclei of irregular and angular shape [168]. Focal disease is mostly sporadic and is associated with a paternally inherited K_ATP_ channel mutation and the loss of the corresponding maternal allele in the focal area [169]. This promotes β-cell proliferation by inducing the expression of insulin-like growth factor 2, inhibiting the tumor suppressor genes H19 and cyclin-dependent kinase inhibitor 1C (CDKN1C) [170]. The Fluorine-18 dihydroxyphenylalanine-positron emission tomography (^18^F–DOPA-PET) scanning can help to localize focal lesion. The principle of ^18^F–DOPA-PET scan is based on the tissue uptake of L-DOPA. Pancreatic islets are able to uptake L-DOPA and convert it to dopamine through DOPA decarboxylase. The uptake of the positron emitting tracer ^18^F–DOPA-PET is increased in β-cells with a high rate of insulin synthesis and secretion compared to unaffected areas (Fig. 2a and c). The sensitivity for detecting focal lesions varies between 88 and 94% with an accuracy of 100% [171]. Intraoperative and postoperative diagnosis of focal congenital HH is based on the presence of adenomatous hyperplasia of β-cells within the focal lesion [172].

### Diffuse form

The diffuse form accounts for 60–70% of all congenital HH cases, and affects all pancreatic β-cells. Morphology of the islets of Langerhans shows the presence of β-cells with abnormally large nuclei (Fig. 2b) [173]. Patients with diffuse congenital HH either have a homozygous recessive or a compound heterozygous mutation in K_ATP_ channel genes [174]. Patients are usually unresponsive to the medical therapy and require a near-total pancreatectomy (95–98% removal).

### Atypical forms

If the pancreatic histology neither fits the focal nor diffuse congenital HH category, this can be defined as atypical form. In these atypical forms some islets show signs of hyperplasia but others appear normal. A previous study reported that some patients with congenital HH have morphological mosaicism including coexistence of two types of islet: large islets with cytoplasm-rich cells and occasional enlarged nuclei and shrunken islets with -cells exhibiting little cytoplasm and small nuclei [170].

## Surgical therapy

Prior to surgery, it is very important to distinguish between diffuse and focal subtypes and localize the lesion in case of focal disease. Recent advances in the molecular genetics of congenital HH and imaging with ^18^F–DOPA-PET/CT scan, have largely contributed to short- and long-term successful management of patients, particularly those with focal disease [173]. While diffuse disease exhibits a diffuse uptake on the ^18^F–DOPA-PET/CT scan, focal form shows a focal uptake (Fig. 2) [175, 176]. Also intraoperative frozen sections are essential both to confirm the diagnosis made based on imaging and to determine whether the suspected area effectively corresponds to a focal lesion and is completely resected [177].

### Surgery of focal disease

**Fig. 3:**
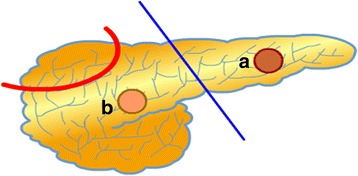
A schematic appearance of pancreatectomy methods for surgery of congenital HH. While for a focal case only limited lesionectomy (**a** and **b**) provide cure without any postsurgical complication, in case of diffuse disease, extensive excision (laparoscopic or open) of a certain part of pancreas may result in continuum of the HH or developing exocrine and endocrine pancreas insufficiency

Detection of paternally inherited mutations in K_ATP_ channel genes and localization of lesion using imaging with ^18^F–DOPA-PET/CT scan provide possibility of completely cure of the disease by limited lesionectomy/partial pancreatectomy, with few or no surgical complications [41, 178, 179]. In proximal lesions in the head and neck of the pancreas, open resection of the lesion with a small rim of surrounding normal pancreatic tissue is carried out and pancreaticojejunostomy is performed to allow drainage of the distal pancreas. Laparascopic distal pancreatectomy is the surgical option for distal lesions (Fig. 3,) [9].

### Surgery for diffuse and atypical disease

Patients with diffuse and atypical disease that are unresponsive to medical treatment usually require extensive surgery (subtotal- or near-total pancreatectomy). Although this might alleviate the recurrence risk of hypoglycaemia, this procedure caries a high risk of developing pancreatic exocrine insufficiency and diabetes which requires life-long pancreatic enzyme replacement and insulin therapy [9, 180–183]. In near-total pancreatectomy, the tail, body, uncinate process and part of pancreatic head are resected, leaving a rim of pancreatic tissue surrounding the common bile duct and along the duodenum [9]. However, despite this radical pancreatectomy some children continue to have HH despite the removal of 95–98% of pancreatic tissue [181]. Diabetes can develop immediately after surgery or later on follow up [180]. These patients who undergo surgical resection should be monitored for glucose metabolism and diabetes [180–183].

## Follow up and outcome of congenital HH

The management of severe HH patients is challenging and requires a multi-disciplinary approach. Integrating the work of a team including clinicians, surgeons, specialized pathologists, geneticists, nurse specialists and dietitians would provide comprehensive management. Early identification and meticulous management of these patients is vital for preventing neurological sequelae. In the studies evaluating the long-term outcome of patients with HH, a high frequency of neurodevelopmental retardation and various neurological disorders including epilepsy and microcephaly have been reported [91, 99, 183]. Besides, the incidence of abnormal neurodevelopment in transient and permanent cases have been found similar [99]. Severity of the disease (based on maximal diazoxide dose) and early presentation (<7 days following birth) were significantly shown to be associated with abnormal neurodevelopment, while gender, underlying genetic etiology or the histopathological form of HH were not related to the neurological outcome [99]. Therefore, neurological development should be closely followed up. Early diagnosis, immediate treatment, definition of the histological subtype, and localization of the focal lesion prior to surgery and regular follow up may improve the outcome of patients.

**Fig. 4:**
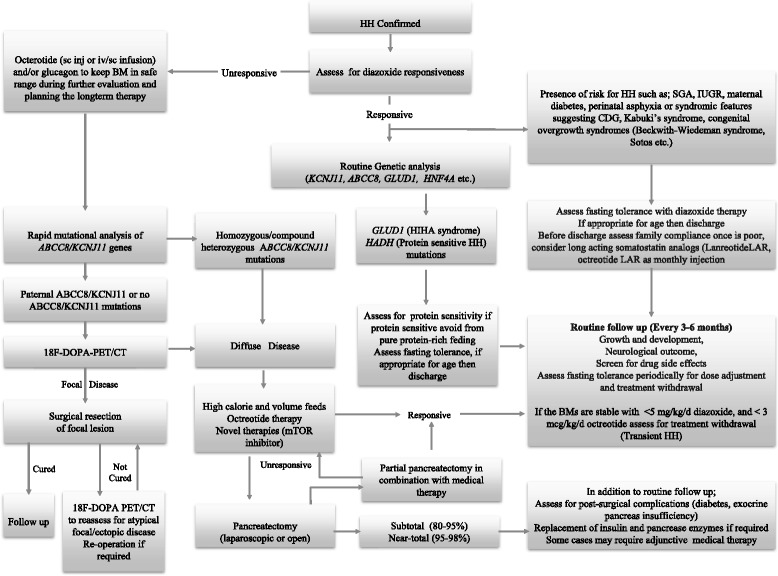
An algorithm for the management of patients with congenital HH

On the other hand, to assess the efficacy of medical therapy, arranging a feeding plan appropriate to the individual fasting tolerance and adjusting the drug doses, repeated fasting tolerance test during follow up may be required [184]. Furthermore, since it is well known that the severity of HH becomes milder overtime, reassessment of fasting tolerance would ensure the cessation of any unnecessary medication [184, 185]. As some forms of congenital HH tend to improve over time, we recommend that patients should be assessed on a regular basis to see if their drugs can be stopped in the hospital setting. As a general guide; if the diazoxide dose is less than 5 mg/kg/day and octreotide is less than 3mcg/kg/day with no hypoglycaemic episdoes, then it might be possible to stop the medications and re-evaluate the patient. Figure 4 outlines management and follow-up of patients with congenital HH. Finally, genetic counselling is recommended for the familial forms of HH.

## Conclusions and future directions

Congenital HH, the most severe cause of hypoglycaemia in the newborn and childhood period, is a complex heterogeneous condition in terms of variable clinical presentation, genetic background, histological subtype and reponse to the medical therapy. The molecular basis of CHI involves defects in key genes (*ABCC8, KCNJ11, GLUD1, GCK, HADH, SLC16A1, HNF1A, HNF4A, UCP2, HK1, PGM1* and *PMM2*) which regulate insulin secretion. Rapid genetic analysis, imaging with ^18^F–DOPA-PET/CT, new medical therapies and surgical techniques have largely contributed to the improvement in the management and outcome of the disease. Although our knowledge and experience about the disease and its management is improving, further research to identify underlying molecular genetics basis of the cases with unknown genetics is required. The discovery of these genes and the mechanisms involved would allow us to identify and develop novel therapeutic options and improve the outcome of this patient group.

## Abbreviations

(18F–DOPA) PET/CT: 18-F-fluoro-L-dihydroxyphenylalanine positron emission tomography
ABCC8: ATP Binding Cassette Subfamily C Member 8
ADP: Adenosine diphosphate
CDG: Congenital disorders of glycosylation
FFA: Free fatty acids
GCK: Glucokinase
GDH: Glutamate dehydrogenase
GLP-1: Glucagon-like peptide-1
GLUD1: Glutamate dehydrogenase 1
GLUT2: Glucose transporter 2
HADH: Hydroxyacyl-CoA dehydrogenase
HH: Hyperinsulinaemic hypoglycaemia
HK1: Hexokinase 1
HNF: Hepatocyte nuclear factors
IUGR: Intrauterine growth restriction
K_ATP_: ATP-sensitive potassium channels
KCNJ11: Potassium Voltage-Gated Channel Subfamily J Member 11
Kir6.2: Inwardly rectifying potassium
LAR: Long-acting release
MCT1: Monocarboxylate transporter
mTOR: mammalian target of rapamycin
PGM1: Phosphoglucomutase 1
PMM2: Phosphomannosmutase 2
PPAR: Peroxisome proliferators-activated receptor
SCHAD: Short chain L-3-hydroxyacyl-CoA dehydrogenase
SGA: Small for gestational age
SLC16A1: Solute Carrier Family 16 Member 1
SUR1: Sulphonlyurea receptor 1
UCP2: Uncoupling protein 2
